# Multifocal transcranial direct current stimulation enhances lower limb jump performance and neuromuscular adaptation in female collegiate basketball players

**DOI:** 10.7717/peerj.20705

**Published:** 2026-02-04

**Authors:** Ruibo Chen, Qingwei Wang, Danyang Li, Binbin Jia

**Affiliations:** 1 Capital University of Physical Education and Sports, Beijing, China; 2 Wuhan Sports University, Wuhan, China

**Keywords:** Multifocal transcranial direct current stimulation, Jump performance, Surface electromyography, Neuromuscular adaptation, Female basketball athletes

## Abstract

**Background:**

Transcranial direct current stimulation (tDCS) has been reported to enhance explosive strength in lower limb skeletal muscles. Nevertheless, findings regarding the impact of tDCS on jump performance remain inconclusive, potentially due to variations in stimulation montage and current intensity. Therefore, we aimed to elucidate the effects of multifocal tDCS on lower limb jump kinetics and neuromuscular adaptation.

**Methods:**

Fourteen female collegiate basketball players were enrolled in a randomized, crossover, controlled trial. Each participant underwent three intervention sessions in a randomized sequence: 2 mA tDCS, 4 mA tDCS, and sham tDCS, all targeting the primary motor cortex (M1). After each stimulation session, countermovement jump (CMJ), squat jump (SJ), drop jump (DJ), and surface electromyography (EMG) data were collected. Statistical analysis was performed using one-way repeated measures ANOVA.

**Results:**

The 4 mA multifocal tDCS condition produced a significant increase in jump height compared to baseline, sham, and the 2 mA condition. Similarly, the concentric impulse was markedly higher in the 4 mA group relative to all other conditions. Relative peak force was significantly improved in the 4 mA group versus baseline, and relative peak power was significantly greater under 4 mA tDCS compared to sham stimulation. The modified reactive strength index (RSImod) was also enhanced considerably following 4 mA tDCS, relative to both baseline and sham conditions. However, EMG analysis indicated that none of the tDCS interventions significantly affected the root mean square (RMS) values of lower limb muscle activation, including the rectus femoris (RF), vastus lateralis (VL), vastus medialis (VM), biceps femoris (BF), semitendinosus/semimembranosus (SEM), medial gastrocnemius (MG), lateral gastrocnemius (GL), and tibialis anterior (TA).

**Conclusion:**

Multifocal anodal tDCS at an intensity of 4 mA significantly improves lower limb jump performance in female collegiate basketball athletes. Integrating multifocal anodal tDCS into routine training regimens may serve as a practical and effective adjunct for enhancing performance in this population.

## Introduction

Basketball is an intensely competitive sport, characterized by frequent physical contact and repeated bouts of jumping, with players averaging 44 jumps per game. Such demands require athletes to possess exceptional lower-limb explosive power. Vertical jump ability is particularly critical for female basketball players and is widely recognized as a key determinant of sport-specific performance (Ziv & Lidor, 2010). However, female athletes exhibit distinct muscle strength characteristics; due to differences in sex and endocrine profiles, women generally have a lower proportion of fast-twitch muscle fibers and a greater reliance on slow-twitch fibers for force production, resulting in reduced strength output compared to their male counterparts. Moreover, lower testosterone levels in females contribute to diminished muscle protein synthesis during resistance training, further limiting strength development (Haizlip, Harrison & Leinwand, 2015). Insufficient strength also predisposes female basketball athletes to a greater risk of injury during high-intensity movements and jumping tasks. Indeed, the incidence of lower limb injuries is significantly higher in female athletes than in males, with the risk of anterior cruciate ligament (ACL) injury reported to be two to ten times greater among female athletes (Zhiguo & Yuanwei, 2016).

The ultimate goal of competitive sports is to push the boundaries of human physical capability. In pursuit of this goal, sports scientists are continually searching for interventions that can yield even marginal improvements in athletic performance (Holgado et al., 2024). Recently, advances in neuroscience have revealed that neuromodulation techniques can regulate the interplay between the nervous system and muscles, thereby improving neuromuscular coordination and efficiency—a subject of growing interest within the field of exercise science (Hornyak, 2017). Among these approaches, transcranial direct current stimulation (tDCS) has emerged as a prominent noninvasive brain stimulation technique. tDCS utilizes low-intensity direct currents delivered *via* scalp electrodes to modulate the excitability of targeted cortical regions. The physiological effects of tDCS are polarity-dependent: anodal stimulation (a-tDCS) increases cortical excitability by depolarizing neuronal membrane potentials, whereas cathodal stimulation (c-tDCS) decreases excitability through hyperpolarization (Machado et al., 2019). Anodal tDCS has been increasingly applied in sports science to enhance attributes such as strength (Lattari et al., 2020), endurance (Vitor-Costa et al., 2015), and balance (Craig & Doumas, 2017). However, its efficacy for improving athletic performance remains equivocal. For instance, Kamali et al. (2019) examined the impact of tDCS on knee extension strength (one-repetition maximum, 1RM) and muscular endurance (strength endurance index, SEI) in bodybuilders. They reported that a 13-minute stimulation protocol targeting the primary motor cortex and temporal cortex elicited improvements of 4.4% in strength and 16.9% in endurance. In contrast, a recent study by Kenville et al. (2024) on the isometric bench press found that 20 min of cerebellar tDCS had no significant effect on upper-limb maximal voluntary contraction (MVC) or rate of force development (RFD). Thus, the impact of a-tDCS on performance remains a topic of debate and is likely influenced by multiple factors, including current intensity, electrode montage, electrode size, and individual variability (Lattari, Oliveira & Márquez, 2022; Lewis, Rattray & Flood, 2025).

Current evidence suggests that placing the anodal electrode over the primary motor cortex (M1) can enhance cortical output, increase corticospinal excitability, and improve athletic performance in elite athletes (Yu et al., 2024). However, the literature on its effects on lower limb explosive power is inconsistent. Some studies have shown that tDCS can increase countermovement jump (CMJ) height (Zhiqiang et al., 2023), whereas others have found no significant benefit (Romero-Arenas et al., 2021). These discrepancies may be attributed to variations in key factors such as current intensity, electrode montage, and task specificity. While conventional protocols typically employ currents of 1–2 mA, recent evidence suggests that higher intensities may be more effective, highlighting the superior efficacy of these higher intensities. For example, Zhang et al. (2025) compared the effects of 1 mA and 3 mA tDCS on lower limb explosive and maximal strength, demonstrating that 3 mA induced significant long-term gains compared to both 1 mA and sham stimulation. Regarding electrode arrangement, traditional montages targeting a single brain region often result in a diffuse electric field distribution with insufficient spatial focality (Masina et al., 2021). Conversely, multifocal tDCS utilizes optimization algorithms to deliver more precise, targeted cortical stimulation (Ruffini et al., 2014). Furthermore, intervention outcomes are modulated by the specificity of the motor task; a recent meta-analysis in athletes indicated that tDCS did not significantly improve gross strength or endurance tasks, but rather yielded small to moderate improvements specifically in visuomotor skills (Maudrich et al., 2022). Moreover, most previous studies have focused on male athletes, with relatively little attention given to well-trained female populations. However, it remains unclear whether higher-intensity protocols can further enhance jump performance, particularly in such athletes. Therefore, the present study aimed to investigate whether high-intensity multifocal tDCS can modulate lower limb muscle recruitment and subsequently enhance jump performance in female basketball players, thereby addressing the limitation in the current literature regarding the comparison of different stimulation intensities. We hypothesized that (1) 4 mA a-tDCS would significantly improve jump performance compared to sham and 2 mA tDCS, and (2) 4 mA a-tDCS would significantly increase lower limb muscle activation relative to sham and 2 mA tDCS.

## Materials & Methods

### Participants

Fourteen female collegiate basketball players were recruited for this study (age: 18.86 ± 1.41 years; height: 175.07 ± 4.76 cm; body mass: 72.86 ± 18.35 kg; Table 1). The sample size was determined by a priori power analysis using G*Power 3.1.9.7. The analysis utilized an F-test for repeated measures ANOVA (within-factors) with the following input parameters: a statistical power (1 − β) of 0.80, a significance level (α) of 0.05, and an effect size of 0.41, based on previous findings (Codella et al., 2021). While the calculation indicated a minimum requirement of 10 participants, we recruited 14 participants to account for potential attrition. Recruitment was conducted *via* advertisements posted on WeChat. Inclusion criteria were: (1) age between 18 and 25 years; (2) no history of significant injuries or neurological disorders; and (3) at least three years of specialized basketball training. Exclusion criteria included: (1) sports injuries such as ligament tears, fractures, or joint dislocations within the past six months; (2) cardiovascular, neurological, or other conditions contraindicating tDCS; and (3) use of medications or supplements in the three months preceding the study (Villamar et al., 2013). To minimize confounding variables, participants were instructed to abstain from strenuous exercise, alcohol, and caffeine for 24 h before each experimental session and to ensure adequate sleep the night before testing. All participants provided written informed consent after receiving a detailed explanation of the study procedures. The protocol was approved by the Ethics Committee of Capital University of Physical Education and Sports (2025A014), and all procedures adhered to the ethical standards outlined in the Declaration of Helsinki. Additionally, this study has been prospectively registered in the Chinese Clinical Trial Registry (ChiCTR2500104305).

**Table 1:** Baseline characteristics of study participants.

Variables	(*n* = 14)
Age (years)	18.86 ± 1.41
Height (cm)	175.07 ± 4.76
Weight (kg)	72.86 ± 18.35
BMI	23.8 ± 6.21
Exercise seniority (years)	7.42 ± 2.43

### Experimental procedure

**Figure 1:**
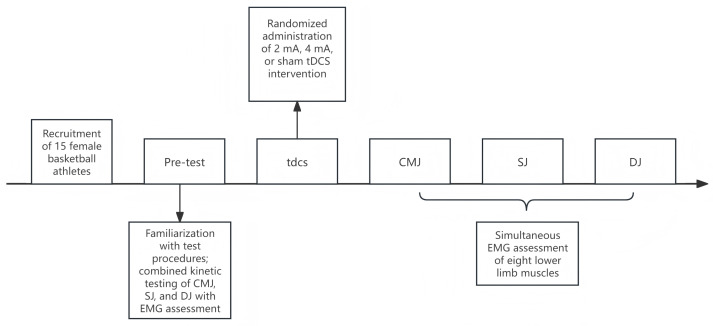
Overview of the study design. a-tDCS, anodal transcranial direct current stimulation; CMJ, countermovement jump; SJ, squat jump; DJ, drop jump; EMG, electromyography.

A randomized, single-blind, crossover design was employed. The order of interventions was randomly assigned to each participant using http://www.randomizer.org. Each participant completed four laboratory visits. Before each experimental session, a standardized warm-up was performed, comprising 5 min of jogging and 5 min of dynamic stretching, supervised by the same researcher to ensure consistency. During the initial visit, participants were instructed in correct movement techniques and completed baseline assessments. In the subsequent three visits, participants received, in randomized order, one of three interventions: 2 mA a-tDCS, 4 mA a-tDCS, or sham tDCS, all targeting the M1 area. Following each stimulation session, data were collected for countermovement jump (CMJ), squat jump (SJ), drop jump (DJ), and surface electromyography (EMG) (see Fig. 1). After each session, participants completed an adverse events questionnaire (Brunoni et al., 2011). Sessions were separated by a one-week washout period to prevent carryover effects. Throughout the study period, participants maintained their regular specialized training regimen (three sessions per week) but were instructed to abstain from additional resistance training for 24 h before each test. All assessments were conducted in a climate-controlled laboratory (temperature: 24–26 °C; relative humidity: 40–50%). To minimize the confounding influence of circadian variations on muscle strength, all testing sessions were scheduled between 13:00 and 17:00 p.m.

### Multifocal tDCS protocol

**Figure 2:**
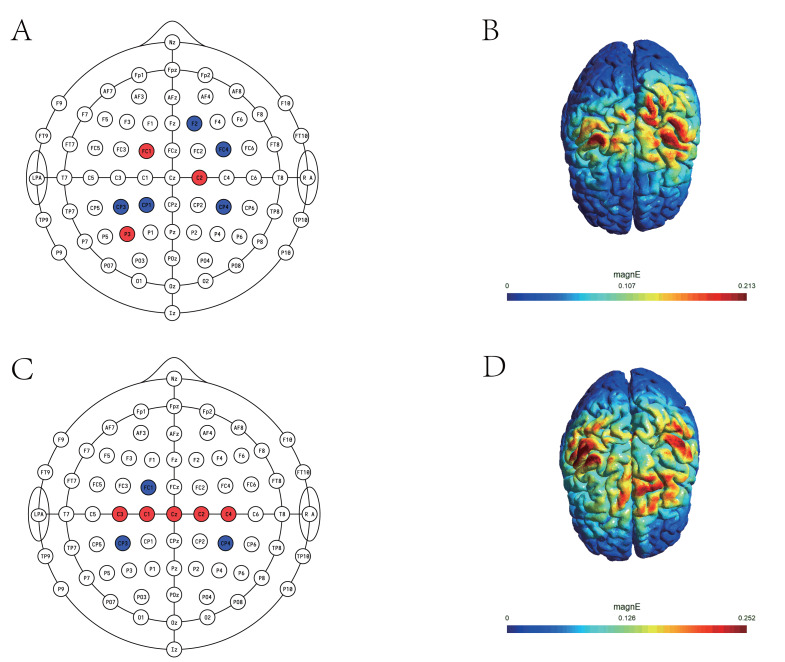
Multifocal tDCS montage. (A) 2 mA montage; (B) electric field distribution on the cortical surface for the 2 mA condition; (C) 4 mA montage; (D) electric field distribution on the cortical surface for the 4 mA condition. Red indicates anodes, and blue indicates cathodes.

Transcranial direct current stimulation (tDCS) was administered using a NeuSen tES device (Shanghai, China), powered by a battery and delivering current through eight circular Ag/AgCl gel electrodes (each 3.14 cm^2^ in area). The maximum current per electrode was 2 mA, and the total current delivered did not exceed 4 mA. The multifocal electrode montage was designed using SimNIBS 4.1 (DTU, Denmark) to optimize the electric field in the bilateral primary motor cortex (M1), based on the international 10–10 EEG system and the Colin 27 brain template. Tissue conductivities were set according to established values for healthy adults: scalp, 0.465 S/m; skull, 0.010 S/m; cerebrospinal fluid, 1.650 S/m; white matter, 0.126 S/m; gray matter, 0.276 S/m (Huang et al., 2013). The finite element method was used to solve the Laplace equation, and both electrode placement and current distribution were optimized to maximize the average electric field in the target region for both 2 mA and 4 mA conditions (Saturnino et al., 2019). The bilateral primary motor cortex (M1) was defined as the target region. For the 2 mA montage, 3 anodes were positioned at P3, FC1, and C2, while 5 cathodes were placed at F2, FC4, CP3, CP1, and CP4, yielding a maximum electric field intensity of 0.213 V/m. For the 4 mA montage, 5 anodes were located at C1, C2, C3, C4, and CZ, and 3 cathodes at CP3, CP4, and FC1, producing a maximum field intensity of 0.252 V/m (see Fig. 2 and Table 2). Sham stimulation was used in either montage, but current was applied for only 60 s (with a 30-s ramp-up and ramp-down), followed by no current for the remainder of the 20-min session. Before stimulation, electrodes were inserted into a neoprene cap and secured in place. The conductive gel was applied to both electrodes and stimulation sites to minimize skin impedance and facilitate effective current delivery. During stimulation, participants remained seated at rest without performing any tasks.

### Lower limb jump testing protocol

A three-dimensional force platform (KISTLER, Switzerland) was used to measure key kinetic variables for the CMJ, SJ, and DJ. Before each test, participants stood motionless with their hands on their hips on the force plate, which was zeroed to account for body weight. Each movement was performed three times, with the best trial recorded, and a 90-second rest between trials. CMJ: Participants performed a rapid, continuous downward and upward movement with their hands on their hips, maintaining trunk stability, aiming to take off and land on the same spot. SJ: From a static squat with knees at 90°, participants jumped vertically on command, without preparatory countermovement, aiming to land on the same spot. DJ: From a 30-cm box, participants stood with hands on their hips, stepped off on command, landed with both feet, and immediately executed a maximal vertical jump.

### Monitoring of lower limb muscle activity

Surface EMG signals were collected from eight right lower limb muscles during the CMJ using a Wave-plus wireless EMG system (Cometa, Italy). Disposable Ag/AgCl electrodes were applied according to SENIAM guidelines, ensuring alignment with muscle fiber orientation. EMG signals were recorded at 2000 Hz. Target muscles included rectus femoris (RF), vastus lateralis (VL), vastus medialis (VM), biceps femoris (BF), semitendinosus (ST), medial gastrocnemius (MG), lateral gastrocnemius (LG), and tibialis anterior (TA). Before electrode placement, the skin was cleansed with alcohol wipes to ensure good adhesion and signal quality. Participants first completed maximum voluntary contraction (MVC) tests for each muscle (5 s per test, with 5-min intervals), after which EMG data were collected during the CMJ.

**Table 2:** Detailed parameters of the multifocal tDCS montage.

2mA Montage		4mA Montage	
Position	Intensity (mA)	Position	Intensity (mA)
P3	0.47	C1	1.02
FC1	0.6	C2	0.72
C2	0.94	C3	0.94
F2	−0.24	C4	0.41
FC4	−0.11	CZ	0.9
CP3	−0.35	CP3	−1.18
CP1	−1	CP4	−1
CP4	−0.3	FC1	−1.81

### Data processing

Ground reaction force (GRF) during jumps was collected and analyzed using MARS software (Kistler, Switzerland). The following kinetic parameters were calculated:

The **jump height** (*H*, cm) was computed from the take-off vertical velocity (*V*) during the countermovement jump (CMJ), where *g* is the gravitational acceleration (9.81 m/s^2^):

(1) \begin{eqnarray*}\mathrm{H}= \frac{{\mathrm{V}}_{0}^{2}}{2\mathrm{g}} .\end{eqnarray*}

The relative peak power (*Relative Pmax*, W⋅kg^−^^1^) was defined as the ratio of the maximum power (*Pmax*) during the propulsive phase of the CMJ to body mass (*m*):

(2) \begin{eqnarray*}\mathrm{Relative}~{\mathrm{P}}_{\mathrm{max}}= \frac{{\mathrm{P}}_{\mathrm{max}}}{\mathrm{m}} .\end{eqnarray*}

The concentric impulse (I, *Ns*) was calculated as the integral of vertical force (*F*) over the duration (*t_0_* to *t_1_*) of the propulsive phase:

(3) \begin{eqnarray*}\mathrm{I}=\int \nolimits \nolimits _{{\mathrm{t}}_{0}}^{{\mathrm{t}}_{1}}\mathrm{F}(\mathrm{t})\mathrm{dt}.\end{eqnarray*}

The **jump time** (T, *s*) was defined as the time interval between the onset of the CMJ and take-off:

(4) \begin{eqnarray*}\mathrm{T}={\mathrm{t}}_{\mathrm{take_off}}-{\mathrm{t}}_{\mathrm{start}}.\end{eqnarray*}

The **countermovement depth** (D, *cm*) was the maximal downward displacement of the center of mass during the eccentric phase:

(5) \begin{eqnarray*}\mathrm{D}={\mathrm{h}}_{\mathrm{standing}}-{\mathrm{h}}_{\mathrm{lowest}}.\end{eqnarray*}

The **modified reactive strength index (RSImod)** was calculated as:

(6) \begin{eqnarray*}{\mathrm{RSI}}_{\mathrm{mod}}= \frac{\mathrm{H}}{\mathrm{T}} .\end{eqnarray*}

The **eccentric utilization ratio (EUR)** was given by:

(7) \begin{eqnarray*}\mathrm{EUR}= \frac{{\mathrm{H}}_{\mathrm{CMJ}}}{{\mathrm{H}}_{\mathrm{SJ}}} .\end{eqnarray*}

The **reactive strength index (RSI)** for the drop jump (DJ) was computed as:

(8) \begin{eqnarray*}\mathrm{RSI}= \frac{{\mathrm{H}}_{\mathrm{DJ}}}{{\mathrm{t}}_{\mathrm{contact}}} .\end{eqnarray*}

Surface EMG data were analyzed using EMG and Motion Tools (Cometa, Italy), which included DC offset removal, band-pass filtering (20–450 Hz, fourth-order Butterworth), full-wave rectification, and a moving average (50 ms window). The period of muscle contraction was identified, and RMS values were calculated (Avdan, Onal & Smith, 2023). The RMS obtained during MVC for each muscle was defined as RMSmvc. The mean RMS during the CMJ was normalized as follows:

(9) \begin{eqnarray*}\mathrm{RMS}= \frac{\text{Mean RMS}}{{\mathrm{RMS}}_{\mathrm{MVC}}} \times 100\%.\end{eqnarray*}

### Data analysis

Statistical analyses were performed using SPSS 26.0 (IBM Corp., Armonk, NY, USA). All data are presented as mean ± standard deviation (SD). The Shapiro–Wilk test was used to assess data normality, and Levene’s test was applied to verify homogeneity of variance. A one-way repeated measures ANOVA was conducted to investigate the impact of the intervention (2 mA, 4 mA, and sham) on lower limb jump kinetics and EMG outcomes. Mauchly’s test of sphericity was performed; when the assumption was violated, the Greenhouse–Geisser correction was used. Bonferroni correction was applied for *post hoc* multiple comparisons. Additionally, a Chi-square test was performed to assess the incidence of adverse events associated with tDCS. For the ANOVA results, effect sizes were reported as ${\eta }_{\mathrm{p}}^{2}$, with thresholds defined as small (${\eta }_{\mathrm{p}}^{2}~\geq $ 0.01), medium (${\eta }_{\mathrm{p}}^{2}~\geq $ 0.06), and large (${\eta }_{\mathrm{p}}^{2}~\geq $ 0.14). For pairwise comparisons, effect sizes were calculated using Cohen’s d and interpreted according to the following criteria: <0.2 (trivial), 0.2–0.5 (small), 0.5–0.8 (moderate), and >0.8 (large) (Cohen, 1988). The significance level was set at α = 0.05.

## Results

### Lower limb jump performance

All 14 participants completed every experimental session, with no dropouts observed. No notable adverse events or potential risks were reported throughout the study. Adverse events associated with each condition are presented in Table 3. Overall, the stimulation protocols were well-tolerated, and no serious adverse events were reported. There were no significant differences in the incidence of adverse effects among the 4 mA, 2 mA, and sham conditions (*p* > 0.05).

**Figure 3:**
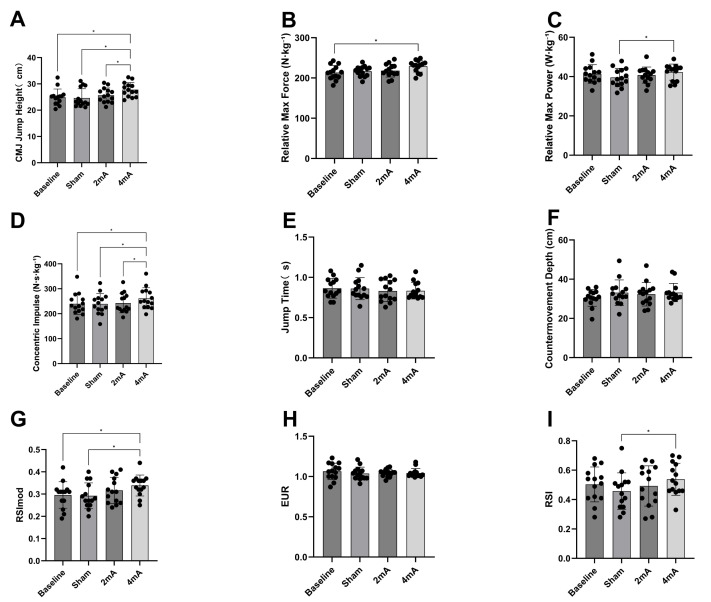
Changes in lower-limb jump kinetic variables following multifocal tDCS intervention. Measurement results are presented as mean (bars) ± standard deviation (error bars) for (A) jump height, (B) relative peak force, (C) relative peak power, (D) concentric impulse, (E) jump time, (F) countermovement depth, (G) RSImod, (H) EUR, and (I) RSI. Significant differences between groups are indicated by asterisks (*, *p* < 0.05).

One-way repeated measures ANOVA revealed a significant effect of the intervention on CMJ jump height (F(3, 39) = 15.23, *p* < 0.001, ${\eta }_{p}^{2}=0.54$). *Post hoc* analyses indicated that the 4 mA group (27.77 ± 2.71) demonstrated significantly greater jump height compared to baseline (24.91 ± 3.12, CI [1.49–4.23], *p* < 0.001 Cohen’s *d* = 0.93), sham (24.63 ± 3.52, CI [1.21–5.06], *p* = 0.001, Cohen’s *d* = 1.03), and the 2 mA condition (25.67 ± 2.75, CI [1.13–3.06], *p* < 0.001, Cohen’s *d* = 0.68). Concentric impulse was also significantly influenced by the intervention (F(3,39) = 13.3, *p* < 0.001, ${\eta }_{p}^{2}$ = 0.5), with the 4 mA group (261.65 ± 42.57) showing significantly higher values than baseline (239.91 ± 42.67, CI [8.84–34.65], *p* = 0.001, Cohen’s *d* = 0.52), sham (239.07 ± 41.9, CI [9.94–35.22], *p* = 0.001, Cohen’s *d* = 0.54), and 2 mA (242.87 ± 39.44, CI [9.7–27.86], *P* < 0.001, Cohen’s *d* = 0.45). For peak concentric force, a significant main effect was observed (F(3,39) = 4.21, *p* = 0.01, ${\eta }_{p}^{2}$ = 0.24), with *post hoc* testing revealing that only the 4 mA group (229.15 ± 14.58) had a significant increase compared to baseline (213.26 ± 17.6, CI [1.59–30.18], *p* = 0.02, Cohen’s *d* = 1.03). Relative maximum power also differed significantly among conditions (F(3,39) = 7.79, *p* < 0.001, ${\eta }_{p}^{2}$ = 0.37), with the 4 mA group (42.28 ± 4.1) exhibiting markedly higher values than the sham group (39.55 ± 4.65, CI [0.92–4.54], *p* = 0.003, Cohen’s *d* = 0.21). The modified reactive strength index (RSImod) was significantly affected by the intervention (F(3,39) = 6.68, *p* = 0.001, ${\eta }_{p}^{2}$ = 0.34); RSImod in the 4 mA group (0.33 ± 0.04) was significantly higher than both baseline (0.29 ± 0.05, CI [0.005–0.08], *p* = 0.02, Cohen’s *d* = 0.76) and sham (0.29 ± 0.05, CI [0.01–0.07], *p* = 0.002, Cohen’s *d* = 0.82) Fig. 3.

No significant effects of the intervention were found for CMJ take-off time (F(3,39) = 0.88, *p* = 0.45, ${\eta }_{p}^{2}=$0.06), countermovement depth (F(3,39) = 1.78, *p* = 0.16, ${\eta }_{p}^{2}$ = 0.12), or eccentric utilization ratio (EUR; F(3,39) = 0.60, *p* = 0.61, ${\eta }_{p}^{2}=0.04$). Regarding the reactive strength index (RSI) during the drop jump, a significant main effect was observed (F(3,39) = 3.71, *p* = 0.019, ${\eta }_{p}^{2}$ = 0.22). *Post hoc* comparisons showed that the 4 mA group (0.53 ± 0.1) had a significantly higher RSI than the sham group (0.45 ± 0.12, CI [0.02–0.13], *p* = 0.005, Cohen’s *d* = 0.65).

**Table 3:** Comparison of adverse effects among sham, 2 mA, and 4 mA tDCS conditions.

	Sham	2 mA	4 mA	*P*
Headache	1 (7.1)	2 (14.3)	3 (21.4)	0.85
Scalp pain	2 (14.3)	6 (42.9)	8 (57.1)	0.06
Tingling	5 (35.7)	8 (57.1)	8 (57.1)	0.42
Itching	4 (28.6)	8 (57.1)	8 (57.1)	0.21
Sleepiness	5 (35.7)	3 (21.4)	4 (28.6)	0.9

### Lower limb electromyography

Analysis of variance showed that the intervention did not produce significant changes in the normalized RMS (%MVCmax) of any monitored lower limb muscle during the CMJ (Table 4). Specifically, no significant differences were detected for the vastus medialis (VM; F(3, 39) = 2.47, *p* = 0.07, ${\eta }_{p}^{2}=0.16$), rectus femoris (RF; F(3, 39) = 1.38, *p* = 0.26, ${\eta }_{p}^{2}=0.09$), vastus lateralis (VL; F(3, 39) = 1.11, *p* = 0.35, ${\eta }_{p}^{2}=0.07$), semitendinosus (ST; F(3, 39) = 0.72, *p* = 0.54, ${\eta }_{p}^{2}=0.05$), biceps femoris (BF; F(3, 39) = 0.29, *p* = 0.83, ${\eta }_{p}^{2}=0.02$), medial gastrocnemius (MG; F(3, 39) = 2.08, *p* = 0.17, ${\eta }_{p}^{2}=0.13$), lateral gastrocnemius (LG; F(3, 39) = 0.96, *p* = 0.41,${\eta }_{p}^{2}=0.06$), or tibialis anterior (TA; F(3, 39) = 1.02, *p* = 0.39, ${\eta }_{p}^{2}=0.07$).

**Table 4:** Changes in lower-limb electromyographic variables following multifocal tDCS intervention.

Muscle	Baseline	Sham	2 mA tDCS	4 mA tDCS	F	*p*
VM	81.24 ± 25.25	104.2 ± 45.18	97.16 ± 35.51	106.4 ± 40.62	2.47	0.07
RF	70.06 ± 19.81	93.75 ± 44.81	80.54 ± 45.28	76.78 ± 27.34	1.38	0.26
VL	106.1 ± 58.55	114.9 ± 43.87	127.1 ± 47.85	102.9 ± 29.06	1.11	0.35
ST	23.76 ± 10.31	34.05 ± 35.24	26.26 ± 14.52	27.19 ± 18.89	0.72	0.54
BF	33.13 ± 23.40	40.45 ± 33.94	37.70 ± 20.79	34.67 ± 21.44	0.29	0.83
MG	59.58 ± 15.99	78.80 ± 32.86	63.62 ± 22.00	75.75 ± 34.53	2.08	0.17
LG	84.72 ± 37.36	95.62 ± 47.11	94.46 ± 53.72	105.8 ± 54.72	0.96	0.41
TA	39.48 ± 7.98	46.23 ± 14.63	43.15 ± 12.15	45.49 ± 15.07	1.02	0.39

**Notes.**

Data are presented as mean (standard deviation). RMS values are expressed as the percentage of the maximum voluntary contraction RMS (%MVC) for each participant.

VM vastus medialis RF rectus femoris VL vastus lateralis ST semitendinosus/semimembranosus BF biceps femoris MG medial gastrocnemius LG lateral gastrocnemius TA tibialis anterior

## Discussion

The primary aim of this study was to evaluate the effects of multifocal tDCS on lower limb jump kinetics and muscle recruitment in female basketball athletes. The findings indicate that, compared to sham and 2 mA multifocal tDCS, the 4 mA multifocal tDCS protocol significantly improved lower limb explosive performance—including vertical jump height, relative peak force, relative peak power, concentric impulse, and reactive strength index—without increasing lower limb muscle recruitment.

### Effects of multifocal tDCS on jump performance

Our results confirm the initial hypothesis of the study. In contrast to most previous research, which has primarily focused on male participants, this investigation targeted female basketball athletes and employed high-intensity multifocal tDCS. The findings provide compelling evidence that a 4 mA tDCS protocol, as opposed to conventional 2 mA stimulation, can significantly enhance lower limb jump performance in female basketball players. A plausible mechanism underlying this effect is that anodal tDCS (a-tDCS) increases corticospinal excitability by depolarizing neuronal membrane potentials. The efficacy of this process depends on the alignment between current flow and axonal orientation (Stagg & Nitsche, 2011). Recent studies have demonstrated that tDCS can enhance motor cortex excitability (Qi et al., 2024). Such changes in cortical or corticospinal excitability may improve strength-related indices by increasing the frequency and synchronicity of motor unit recruitment, thereby facilitating enhanced short-term explosive force production (Fadiga et al., 2004). These findings are consistent with previous work by Lattari et al. (2020), Codella et al. (2021), and Zhiqiang et al. (2023). Lattari et al. (2020) were among the first to investigate the effects of tDCS on lower limb jump performance, reporting that a-tDCS (2 mA, anode over M1, cathode over the right supraorbital area) increased jump height by 11%. Codella et al. (2021) applied the Halo Neurostimulation device for bilateral M1 stimulation and observed an acute increase of 2.7 cm in jump height compared to sham. Zhiqiang et al. (2023) found a 3.7 cm improvement in CMJ height following bilateral anodal M1 tDCS (2 mA) relative to sham. In contrast, Romero-Arenas et al. (2021) failed to observe significant effects of tDCS on jump height, which may be attributed to the functional specificity of the stimulated brain region. Specifically, Romero-Arenas et al. (2021) applied anodal stimulation to the left dorsolateral prefrontal cortex (DLPFC), a region more closely associated with cognitive, fatigue, and inhibitory control processes and less directly involved in neuromuscular drive (Filipas et al., 2020). By contrast, the primary motor cortex (M1) is the principal region for motor control, governing not only muscle force but also the direction and trajectory of movement (Kakei, Hoffman & Strick, 1999). Thus, anodal tDCS targeting M1 is more likely to maximize the recruitment of strength-related neural networks at the physiological level (Radel et al., 2017).

Importantly, only high-intensity (4 mA) tDCS produced significant improvements in jump performance compared to the 2 mA protocol. A plausible explanation for this finding is the linear dose–response relationship inherent to tDCS. Higher current intensities have been shown to not only enhance cerebral perfusion but also modulate the functional connectivity of sensorimotor networks, thereby driving observable changes in behavioral outcomes (Shinde et al., 2021). Early studies typically employed 1–2 mA stimulation, and many failed to detect significant enhancements in strength or power (Maudrich et al., 2022). Emerging evidence suggests that higher tDCS intensities yield superior ergogenic effects. For instance, Zhang et al. (2025) compared the impact of 1 mA, 2 mA, and 3 mA stimulation on lower limb strength, reporting that 3 mA tDCS significantly increased both CMJ height and peak torque. Furthermore, the electrode montage plays a critical role in determining electric field distribution and stimulation efficacy. Conventional studies often employ electrode placements based on TMS hotspots or the EEG 10–20 system, which tend to generate diffuse electric fields with poor spatial focality, failing to achieve precise targeted stimulation (Caulfield & George, 2022). In the present study, we employed optimization algorithms to adjust electrode placement and current distribution specifically for the M1 region. This approach was designed to induce a stronger and more focused electric field within the target area, thereby potentiating the effects of tDCS (Ruffini et al., 2014; Saturnino et al., 2019). Previous findings support this methodological distinction; while Romero-Arenas et al. (2021) utilized a traditional montage and observed no significant improvement in CMJ performance, Liang et al. (2024) demonstrated that multifocal tDCS could significantly enhance cycling endurance in healthy adults. Therefore, the improvements in jump performance observed in the present study may be attributed to the combined effects of high-intensity and multifocal tDCS stimulation of the M1, ultimately leading to increased short-term lower limb muscle power output.

### Effects of multifocal tDCS on lower limb muscle regulation

The activity level of the primary motor cortex is closely associated with the extent of muscle recruitment (Schwartz, 2007), and surface electromyography (sEMG) is commonly used to evaluate neuromuscular function (Li et al., 2021). As a time-domain parameter, the root mean square (RMS) of sEMG reflects muscle activation level, with higher RMS values indicating greater activation (Sun et al., 2022). In the present study, no significant differences in RMS values were observed among the different intervention conditions, suggesting that higher stimulation intensity did not affect muscle recruitment. This finding may relate to inhibitory feedback mechanisms induced by tDCS, which could serve to limit motor cortical output and thus protect the motor system from excessive load (Cogiamanian et al., 2007). Moreover, surface electromyography (sEMG) is subject to inherent limitations, such as signal crosstalk and amplitude cancellation (Farina, Merletti & Enoka, 2004). Consequently, sEMG may lack the sensitivity required to detect subtle modulations in the central recruitment of spinal motor neurons, particularly during high-intensity contractions. This interpretation is consistent with findings by Workman, Fietsam & Rudroff (2020), who applied 4 mA tDCS over the primary motor cortex and found no significant difference in leg muscle sEMG activity compared to sham stimulation. Similarly, Cogiamanian et al. (2007) showed that tDCS over the motor cortex could prolong the isometric contraction duration of the knee extensors without corresponding increases in sEMG-measured muscle activation. However, some studies have reported that tDCS significantly alters muscle RMS values. For instance, Kamali et al. (2019) found that anodal tDCS over the motor cortex increased knee extension performance and enhanced rectus femoris RMS by 5.8%. Such discrepancies may stem from differences in training status, muscle fiber composition, or specific tDCS protocols among participants. The present study involved female collegiate basketball athletes, who may respond differently to tDCS than bodybuilders—who might possess greater neural adaptation capacities and thus be more likely to exhibit sEMG changes. Overall, our findings suggest that multifocal tDCS does not significantly alter muscle recruitment during jumping tasks in female basketball athletes.

The efficacy of tDCS is also contingent upon population-specific characteristics, as athletes and non-athletes may exhibit distinct responses to stimulation. According to the neural efficiency hypothesis, higher cortical efficiency enables individuals to achieve optimal task performance with minimal metabolic cost (Del Percio et al., 2008). Functionally, long-term systematic training endows athletes with robust functional connectivity within neural networks, facilitating the automaticity of movement execution and reducing energy expenditure (Tan et al., 2017; Li & Smith, 2021). Structurally, elite athletes exhibit elevated baseline levels of neural excitability and brain-derived neurotrophic factor (BDNF) concentrations (Yu et al., 2024). Collectively, these factors suggest that athletes may be subject to a “ceiling effect” in neural processing; because their functional brain networks are already highly optimized, conventional or low-intensity tDCS may be insufficient to elicit further gains in neural activity or behavioral performance. This elucidates why the 2 mA protocol failed to produce significant effects in the present study, whereas the 4 mA multifocal a-tDCS yielded marked improvements. It appears that in athletic populations, higher stimulation intensities are requisite to overcome this physiological ceiling and induce further adaptation.

This study has several limitations. First, the relatively small sample size, though determined by power analysis, may restrict the generalizability of the findings. Future studies should include larger and more diverse cohorts, encompassing a broader range of ages and training backgrounds, to enhance the representativeness and applicability of the results. Second, this study focused only on kinetic and surface electromyographic indices, lacking neurophysiological assessments. Future research should incorporate transcranial magnetic stimulation (TMS) to assess changes in cortical excitability and inhibition following tDCS, such as through motor-evoked potentials (MEP) and short-interval intracortical inhibition (SICI), to characterize corticospinal adaptations more precisely. Additionally, this study did not specifically control for menstrual cycle phase, although fluctuations in hormones and neurotransmitters can influence cortical excitability in females (Rudroff et al., 2020). Future work should conduct experiments at defined menstrual phases (*e.g.*, follicular or luteal) to minimize the confounding influence of hormonal variability on neuromodulatory effects. It is crucial to acknowledge that the utilization of distinct montages and stimulation intensities in this study may have recruited disparate brain network connectivity systems, potentially involving the cerebellum or pain modulation pathways, thereby exerting an indirect influence on motor performance. Consequently, the observed performance enhancements cannot be attributed solely to the disparity in current intensity; they may also stem, in part, from the synergistic or inhibitory interactions of distinct neural networks. Future investigations should employ neuroimaging modalities to conduct brain network connectivity analyses, thereby elucidating the specific neural connectivity mechanisms underlying the effects of tDCS. Additionally, the present study did not account for potential variations in participant body mass over the multi-week intervention period. Given that body mass fluctuations can confound jump kinetics (Markovic et al., 2014), future research should incorporate body mass as a covariate to provide a more accurate assessment of intervention effects.

## Conclusions

This study examined the effects of multifocal transcranial direct current stimulation (tDCS) on lower-limb jump kinetics and neuromuscular adaptation. Compared with sham and 2 mA stimulation, 4 mA multifocal anodal tDCS produced significant improvements in key kinetic outcomes among female collegiate basketball players, including jump height, relative peak force, relative peak power, concentric impulse, and the modified reactive strength index (RSImod). By contrast, no significant differences were observed for time to take-off, countermovement depth, eccentric utilization ratio (EUR), or the traditional reactive strength index (RSI). Similarly, indices of lower-limb muscle recruitment did not show clear advantages. In sum, 4 mA multifocal tDCS appears to selectively enhance lower-limb jumping performance and may serve as a practical adjunct to training to improve force output and sport-specific performance in female basketball athletes.

##  Supplemental Information

10.7717/peerj.20705/supp-1 Supplemental Information 1 Raw EMG Data from CMJ and SJ Tests

10.7717/peerj.20705/supp-2 Supplemental Information 2 Jump Performance Data for CMJ and SJ Tests Jump height and other kinetic parameters recorded during the CMJ and squat jump (SJ) tests.

## Additional Information and Declarations

### Data Availability

The following information was supplied regarding data availability: The raw data is available in the Supplemental Files.
